# Toward the Commercialization of Carbon Nanotube Field Effect Transistor Biosensors

**DOI:** 10.3390/bios13030326

**Published:** 2023-02-27

**Authors:** Zhongyu Li, Mengmeng Xiao, Chuanhong Jin, Zhiyong Zhang

**Affiliations:** 1 Hunan Institute of Advanced Sensing and Information Technology, Xiangtan University, Xiangtan 411105, China; 2 Key Laboratory for the Physics and Chemistry of Nanodevices and Center for Carbon-Based Electronics, School of Electronics, Peking University, Beijing 100871, China; 3 Jihua Laboratory, Foshan 528200, China; 4 State Key Laboratory of Silicon Materials, School of Materials Science and Engineering, Zhejiang University, Hangzhou 310027, China

**Keywords:** field effect transistor, carbon nanotube, biosensors, commercialization

## Abstract

The development of biosensors based on field-effect transistors (FETs) using atomically thick carbon nanotubes (CNTs) as a channel material has the potential to revolutionize the related field due to their small size, high sensitivity, label-free detection, and real-time monitoring capabilities. Despite extensive research efforts to improve the sensitivity, selectivity, and practicality of CNT FET-based biosensors, their commercialization has not yet been achieved due to the non-uniform and unstable device performance, difficulties in their fabrication, the immaturity of sensor packaging processes, and a lack of reliable modification methods. This review article focuses on the practical applications of CNT-based FET biosensors for the detection of ultra-low concentrations of biologically relevant molecules. We discuss the various factors that affect the sensors’ performance in terms of materials, device architecture, and sensor packaging, highlighting the need for a robust commercial process that prioritizes product performance. Additionally, we review recent advances in the application of CNT FET biosensors for the ultra-sensitive detection of various biomarkers. Finally, we examine the key obstacles that currently hinder the large-scale deployment of these biosensors, aiming to identify the challenges that must be addressed for the future industrialization of CNT FET sensors.

## 1. Introduction

The rapid and accurate detection of specific biomolecules is essential for medical diagnostics, food safety, and environmental monitoring [1,2,3]. Driven by the concepts of the Internet of Things (IoT), big data, and wisdom medical, next-generation bio-sensing equipment require the core sensing elements to not only be highly sensitive and selective but also to fulfill the requirements of point-of-care (POC) applications [4,5]. To meet the demands of POC devices, sensor technology that is suitable for mass production, capable of accurately sensing the desired biomarkers, and cost-effective in terms of its widespread use is crucial. However, commercial bio-detection techniques, such as optical or magnetic methods, are often time consuming and require the use of bulky equipment, making them unsuitable for POC applications.

Field-effect transistors (FETs) are believed to be ideal platforms for highly integrated and smart bio-sensor chips due to their potential for miniaturization and mass production [6,7,8,9,10]. FET-based biosensors use the basic characteristics of transistors to directly convert and amplify difficult-to-detect biological (chemical) binding activities or ion concentration changes into electronic signals that are easy to detect and proportional to the presence of the target analyte in the test sample [11,12,13,14,15,16]. With the development of nanotechnology, various FET-based biosensors with excellent performance have been developed by incorporating nanomaterials as the semiconducting channel [17,18,19,20,21,22,23,24,25,26,27,28].

**Figure 1:**
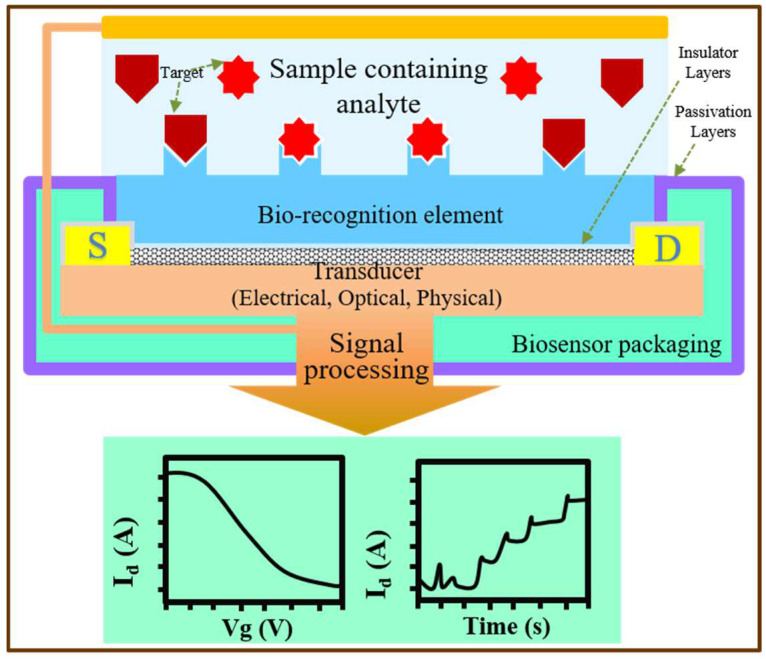
Schematic diagram of the working principle of biosensors based on single-walled carbon nanotube field-effect transistors.

Among the various nanomaterials available, biosensors based on single-walled carbon nanotube field-effect transistors (Bio–CNTFET) detect biomarkers by sensing changes in charge numbers or charge distribution on the nanoscale surface, thereby offering high sensitivity, a small size, and label-free and real-time detection, and making them ideal for the development of POC applications [29,30,31]. As shown in Figure 1, the sensing process of a CNT FET-based biosensor typically involves two steps: the selective capture of target molecules and the transduction of molecular charge information into a current change with FET amplification [11,12,13,14,15,32]. The selectivity of the biosensor is guaranteed by the highly specific biological affinity layer functionalized on the FET’s surface, while sensitivity depends on the electrical gain of the FET, which is related to the intrinsic properties of the channel material and the sensing mechanism of the device structure. For optimal sensor performance, all components of the transistor must be well-designed, including the channel material, contact metal, gate (reference) electrode, and passivation layer. These components must be sensitive enough to detect small changes in target biomolecules and robust enough to maintain stable electrical performance in the harsh physiological environment of the medium.

**Figure 2:**
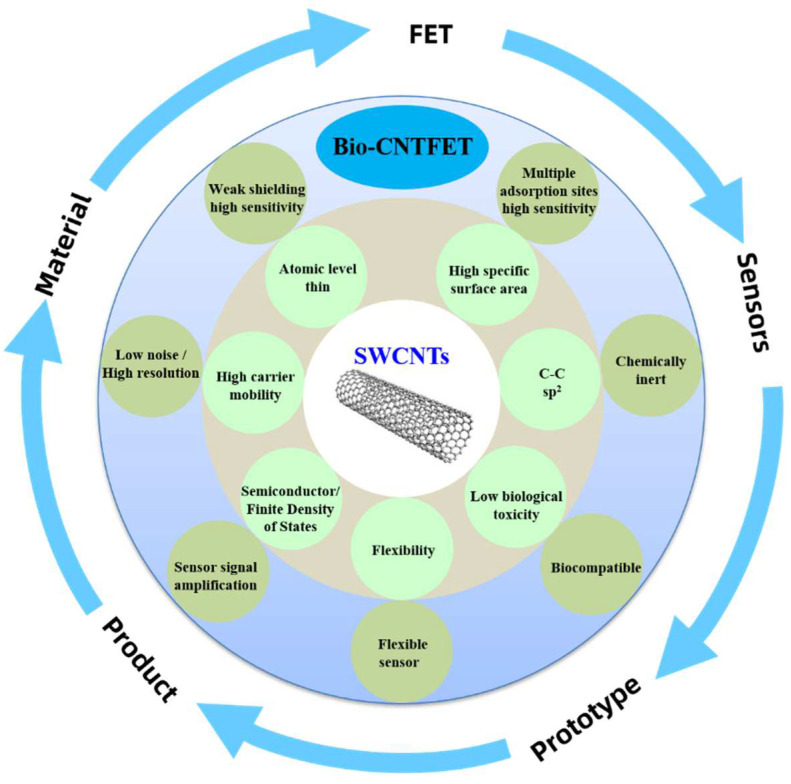
The advantages of using single-walled carbon nanotubes in sensors.

**Figure 3:**
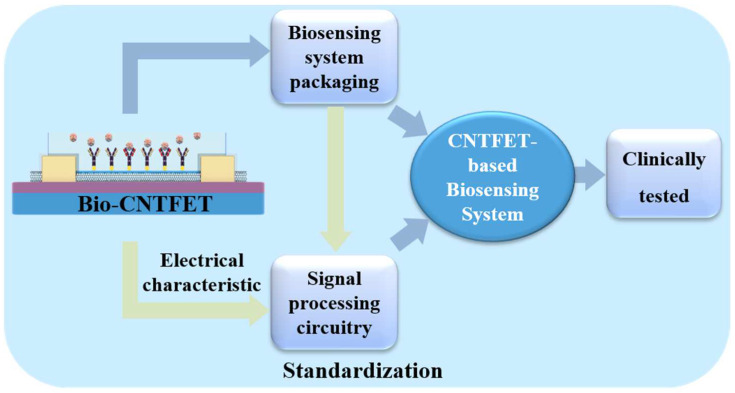
A necessary part of Bio–CNTFET commercialization process.

The key advantage of CNTs lies in their atomic thickness and high electrical mobility, which leads to high current drive capability and superior electrostatic control [20,33,34,35,36,37]. As shown in Figure 2, this makes CNTs a promising material for the fabrication of multifunctional smart biosensor chips, which combine high-performance ICs with highly sensitive electrical sensors. The commercialization of biosensors refers to the application of biosensor technology to the commercial market to meet market demands. This includes the development of reliable and repeatable sensor technology, as well as the implementation of effective marketing strategies to ensure the successful commercialization of the product. After decades of development, the development of Bio–CNTFET technology has progressed from the formation of technical concepts to application schemes at the basic research stage, technical verification in laboratory settings, and system demonstrations in various simulated environments. However, there is still a considerable degree of advancement that must be achieved before they can be applied in practical diagnosis. This review provides a critical overview of the recent developments in the use of CNTs for the highly sensitive detection of biologically relevant molecules from the perspective of practical applications, focusing on material requirements, device fabrication, figures of the metrics of the sensors, package methods, and state-of-art of biomarker detection (Figure 3). Finally, we discuss the future challenges regarding the widespread deployment of Bio–CNTFET from laboratories to practical applications.

## 2. Carbon Nanotube Materials

**Figure 4:**
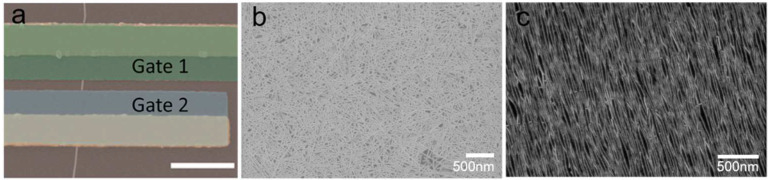
Morphology of single-walled carbon nanotubes. (**a**), Single carbon nanotube [48]. (Copyright © 2015, ACS Nano. (**b**), semiconducting CNT network film. (**c**), Aligned CNT arrays.

Single-walled carbon nanotubes (SWCNTs) present metallic (m-) or semiconducting (s-) properties according to their chirality [38,39]. The adsorption of charged molecules has little effect on the conductivity of metallic CNTs due to their large density of states around the fermi level [40,41,42]. Therefore, CNTs with high semiconductor purity are necessary for the construction of high-performance Bio–CNTFETs [43,44,45,46,47]. There are three types of morphologies for s-CNT materials for electronic applications: chemical-vapor-deposition (CVD)-derived individual tubes, solution-derived network films, and CVD- or solution-derived horizontally aligned CNT arrays (Figure 4). Table 1 comprehensively presents the state-of-the-art performance of different types of CNT materials [48].

Due to their extremely small quantum capacitance, the FETs built on individual s-SWCNTs have shown an ultra-high biomolecular charge sensitivity such that even slight variations in charge number or distribution can induce a substantial change in electrical conductance [49]. The capacity of individual CVD-derived CNT-based FETs has been demonstrated with respect to the detection of protein conformational motion dynamics at the single-molecule level [50,51]. However, the uncontrollability of their locations and electrical properties renders them complicated in terms of device fabrication and unsuitable for the large-scale reproduction of devices in practical applications. Moreover, the small capture cross-section of a single tube and high electrical noise level present large barriers for the sensors’ application in the rapid detection of ultra-low-concentration biomarkers [12,50].

Aligned CNT arrays are favored for electrical sensing applications due to their reduced shielding between tubes and reduced amount of tube-to-tube tunneling compared to network film [47,52,53,54,55,56]. Their film-like morphology also enables the mass production of FET sensor devices with lower baseline electrical noise and improved device yield and reproducibility [57]. There are two methods for preparing CNT arrays: CVD synthesis and solution methods. However, CVD–derived CNT arrays typically exhibit a mixture of metallic and semiconducting ones. While solution-derived CNT arrays hold promise, they still present challenges in terms of uniform coverage on a wafer-scale. The major hindrance to the practical implementation of Bio–CNTFETs is the creation of devices with nearly identical field-effect characteristics at the wafer-level.

At present, the use of wafer-scale uniform semiconducting CNT network films for the construction of FET biosensors holds significant potential for commercial applications [51,58]. In the past, the presence of different electrical properties in raw CNT materials has impeded the widespread application of CNTs in biosensor technology. However, recent advancements in technology have led to rapid improvements in the semiconductor purity of solution-dispersed CNTs, with some samples reaching a purity level of 99.9999%, at which degree not a single metallic CNT can be found among two million CNTs. With the dispersed and purified s-CNT solution, a randomly distributed s-CNT thin film can be obtained via the dip-coating method at the wafer scale, and this solution-dispersed method can easily be scaled up [59,60,61]. The randomly oriented network film ensures homogenous properties and provides a solid foundation for reproducible device fabrication [49,62,63,64,65,66]. The wafer-scale uniform semiconducting CNT network films have proven to be an attractive option for FET biosensors due to their remarkable potential for meeting the requirements for industrialization.

## 3. Device Structures and Working Principles of CNT FETs for Biochemical Sensing

### 3.1. Working Principles of CNT FET for Biochemical Sensing

**Figure 5:**
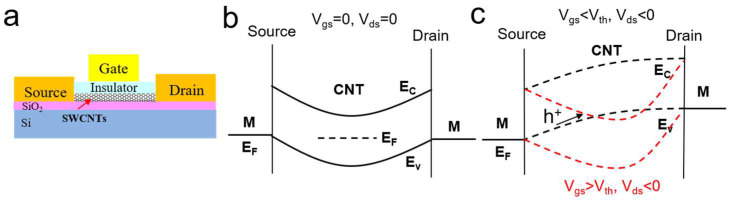
Schematic representation of the device structure of CNTFET. (**a**) The three-electrode configuration in a CNTFET. (**b**,**c**) Device energy band diagram.

SWCNTs are an attractive material for electrical sensing due to their small size (1–2 nm diameter) and low surface state density, which provide excellent electrostatic control and charge sensitivity toward surrounding charge variations [20,33,34,35,36,37]. Figure 5a shows the schematic diagram of a top-gated CNTFET. Since there is no fermi pining between the CNTs and contact metal due to the dangling bond-free surface of the CNTs, a Schottky contact can be formed, and the contact resistance can be modulated by using different work function metals as contacts. The gate electrode modulates the bending of the energy band and relative positions of the Fermi energy levels in the carbon nanotube channels through a vertical electric field, thereby modulating the potential barriers faced by the electrons and holes in the source and drain electrodes [40,41,42]. A horizontal electric field between the source and drain drives the charge carriers (electrons or holes) through the channel to produce a drain-source current. As presented in Figure 5b,c, p-type Ohmic contact devices with excellent performance can be obtained by using high-work-function metals with good affinity towards CNTs [67,68,69,70,71]. A positive voltage applied to the gate causes the energy barrier for holes to increase, thereby turning the CNTFET off (Figure 5c). The response of a CNTFET-based biosensor arises from changes in the energy band caused by biomolecules’ modulation of the gate, contact, or the semiconductor channel directly.

### 3.2. Device Structures

**Figure 6:**
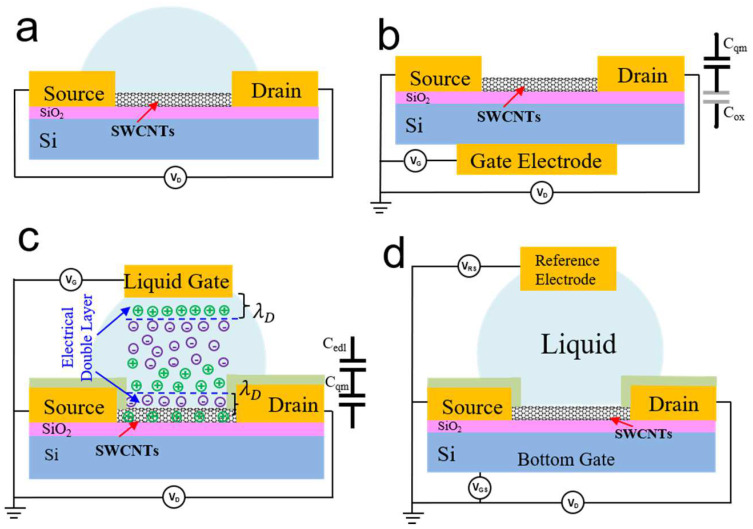
Three device structures of Bio–CNTFET. (**a**) Two-Electrode sensor. (**b**) Back-gate control device structure. (**c**) Electrolyte-gated control device structure. The electric double layer at the CNT–liquid interface acts as a gate insulator. A reference electrode, such as a Ag/AgCl wire, is used as the gate electrode. The electrical double layer at the CNT–liquid and liquid–gate interfaces is the source of geometric capacitance (C_ox_). The quantum capacitance (C_qm_) and geometric capacitance of carbon nanotubes constitute an in-series relationship. (**d**) Electrolyte trench isolation type.

When integrating SWCNTs into electronic biosensors, several sensing mechanisms, such as electrostatic gating, charge scattering, Schottky barrier modulation, or capacitance modulation, take effect depending on the type of device structure design that has been employed [60,61,62]. The configurations of Bio–CNTFET devices typically include a two-end device, a back-gate device, an electrolyte-gated device, and a dual-gate device, as depicted in Figure 6. The electrolyte-gated devices undoubtedly constitute the most interesting platform. Electrolyte-gated devices are divided into electrolyte-gated-channel-exposed devices and electrolyte-gated channel-isolated devices. The interaction of target analytes with the biometric element leads to changes in the electrical properties of CNTs, such as their resistance or field-effect behavior, which can be detected by measuring the output current [72].

Several studies have utilized two–end device structures to detect changes in the resistance or conductance of single CNTs or CNT networks in the presence of DNA (Figure 6a) [21,73,74]. However, this approach has limitations in terms of sensitivity and versatility due to the lack of control and regulation of device performance. The back–gated Bio–CNTFET (Figure 6b) is a relatively simple device with a gate and source drain on different sides of the silicon substrate, thus minimizing damage to the channel layer material from the multiple processing steps [75,76,77,78]. The channel conductivity can be modulated through the field effect by applying the potential to the highly conductive silicon substrate in a large range of back-gated voltage (V_GS_) [79,80]. The gate insulator’s oxide capacitance (C_ox_) and the carbon nanotubes’ quantum capacitance (C_qm_), which together form the geometric capacitance, determine the device’s characteristics [7,8,81,82,83]. Star et al. reported that CNTFET can specifically recognize target DNA sequences after immobilized oligonucleotide synthesis [84]. However, back-gate devices are designed to measure device performance in dry environments by applying a gate voltage to the substrate, as presented in previous research [23]. Back-gated FETs are not appropriate for real-time measurements in liquid environments due to the lack of electrode passivation. The presence of liquid can interfere with the electrical signals required for proper FET function and lead to inaccurate readings. Moreover, the liquid can trigger the corrosion of the FETs and result in unstable FETs, leading to unreliable measurements. A back-gated Bio–CNTFET has lower gate control efficiency and sensitivity compared to liquid gate structures and cannot perform real-time measurements in liquid environments [85], thereby hindering its potential for high-performance biosensing applications.

Another typical configuration involves the use of a liquid gate with a reference electrode (Figure 6c) [86] and a dual-gate Bio–CNTFET (Figure 6d). The electrolyte-gated Bio–CNTFET (Figure 6c) applies gate voltage to the solution being tested between the source and drain, using the solution medium as the gate layer of the FET [86]. A dual-gate Bio–CNTFET can amplify small biological signals by several orders of magnitude using capacitive coupling effects occurring between the top and bottom gates of the channel, thereby offering better signal-to-noise ratios compared to single-gate counterparts. However, most of the research on dual-gate Bio–CNTFETs focuses on the theoretical model, and the difficulty of device preparation is relatively high, which limits its wide application [87]. In an exposed–channel liquid-gate structure (Figure 6c), the geometric capacitance is formed by the double layer capacitance (C_edl_) in series at the CNT–liquid and liquid–gate interfaces [86,88]. The quantum capacitance (C_qm_) of carbon nanotubes is in series with the geometric capacitance and dominate over a wide range of voltages, thereby overcoming hysteresis [7,8]. However, due to the active CNT semiconductor channel directly exposed to the electrolyte, the sensors’ working principles become so complex that four kinds of interaction take effect at the same time, as described previously [89]. The competing effect on the channel conduction change may reduce the sensor’s response and sensitivity. Furthermore, direct contact between the electrolyte and the channeled material leads to ion adsorption on the surface of the channel material via repetitive ionic doping and de-doping, thus decreasing the sensor’s cyclability and lifetime.

The electrolyte-gated-isolated-channel Bio–CNTFET (also known as a floating-gated Bio–CNTFET) avoids the adverse effects conferred by electrolyte solutions on channel materials by introducing a dielectric layer between the electrolyte and the channel, as shown in Figure 6d. The source and drain electrodes are passivated by an insulator layer to prevent current leakage and electrochemical reactions, thereby eliminating the Schottky barrier modulation effect. The charge variation induced by the hybridization of the target biomolecules on the gate is capacitively coupled with the semiconductor channel, leading to a threshold voltage shift in the FET. Liang et al. [90] introduced an ultra-thin, high-k yttrium oxide dielectric layer between the CNT channel and the electrolyte environment to avoid the influence of competitive and non-electrostatic modulation, e.g., a scattering effect. In addition, 0.6 nm Au nanoparticle layers were assembled on it as linkers to connect –HS (Mercapto group) bio-receptors. The Y_2_O_3_ dielectric layer provides high gate efficiency and an excellent interface with CNTs, resulting in a highly efficient electrostatic gating effect on the channel caused by biomolecules. The total capacitance comprises the double layer capacitance, the dielectric capacitance of C_ox_, and the quantum capacitance in series [91]. Due to the high capacitance of the electrical double layer and the ultra-thin high-k oxide layer, the electrostatic gating effect on the channel induced by the biomolecules is highly efficient, which ultimately enables the attainment of a highly sensitive sensor platform. Adopting mature CNT–based CMOS manufacturing technologies can help improve the stability and uniformity of Bio–CNTFET devices and promote their future integration and practical applications in biosensors. The physical model of this device configuration can be simplified to electrostatic gating, thereby aiding the development of an analytical model for further improved designs.

## 4. Relationship between CNT FET Performance and Biosensor Performance

The interaction between the probe and target molecule in a Bio–CNTFET sensor follows the Langmuir isotherm model [92].

(1) $$Q_{absorb}=\frac{Q_{max\;}\cdot \;c}{\frac{1}{K}+c}\;.\;$$

The maximum charge of all the active probe molecules that are hybridized to the target molecules is represented by *Q_max_* (*e*). *c* (M) is the concentration of the target molecule. The equilibrium constant of the target molecules is denoted by *K* (M^−1^) = *k*_1_/*k*_−1_, where k_1_ and *k*_−1_ are the association and dissociation constants, respectively. This equation can be further Taylor expanded around *Q_max_*/2 to the following form:

(2) $$Q_{absorb}=\frac{Q_{max}}{4{log}_{10}e}\left[{log}_{10}c+{log}_{10}\left(e^{2}K\right)\right]\;.$$

This means that the sensor response can be expressed as a linear function of a logarithmic concentration in a certain concentration range. Details of the derivative process can be found in [93].

In the linear region of the FET with a small source-drain bias voltage, we have *V_ds_* << *V_gs_ − V_th_*.

(3) $$I_{ds}=\frac{W}{L}uC_{ox}(V_{gs}-V_{th}-\frac{V_{ds}}{2})V_{ds}\approx g_{m}\left(V_{gs}-V_{th}\right)\;$$

In the Equation above, *L* and *W* represent the channel length and width of the transistor. μ represents the channel mobility. In the linear region of the FET with a small source-drain bias voltage, the transconductance is constant. *g_m_* (*s*) is the derivative of the source-drain current with respect to gate voltage at a fixed small source-drain bias, which reflects the speed of the activation of the device: $g_{m}=\frac{\partial I_{ds}}{\partial V_{gs}}{|}_{V_{ds}=const}=\frac{W}{L}uC_{ox}V_{ds}$.

There are three typical definitions of a FET sensor’s response.

1. Voltage shift (Δ*V_th_*)

This definition more directly reflects the effect of the biomolecular charges on device performance. In some cases, Δ*V_th_* = △*I*/*g_m_* [94] is used as a calibration parameter for the sensor response derived from [85]. △*V_th_* is the equivalent gating voltage (potential) induced by the biomolecules. △*I* is the absolute current change before and after the target molecules’ interaction. *g_m_* is the transconductance of the FET devices.

For the chemical gating effect that dominates FET biosensors, including the FG CNT FET biosensors, the equivalent voltage shift can be expressed as

(4) $$\Delta V_{th}=\frac{Q_{absorb}}{C_{total}}=\frac{Q_{max}}{C_{ox}\cdot C_{qm}/(C_{ox}+C_{qm})}\frac{c}{c+\frac{1}{K}}\;.$$

where *c* is the total capacitance contributed by double layer capacitance, the dielectric capacitance of *C_ox_*, and the quantum capacitance, as mentioned before. A smaller degree of quantum capacitance of a one-dimensional CNT material leads to a higher shift in *V_th_*, allowing one to derive the relationship between the sensor response and the FET parameters from the output characteristics.

In actual detection, it is necessary to scan the transfer characteristics of devices to determine the threshold voltage, or to use a constant current input method to convert the response to a voltage change, which adds complexity to the signal acquisition and processing circuit.

2. Absolute current change (Δ*I*)

Acquiring the change in current in a constant voltage mode is easier and more convenient. Based on the Langmuir isothermal model, the absolute current change can be written as follows:

(5) $$\Delta I=G_{L}\times \Delta V_{A}\approx g_{L}V_{ds}\times \frac{\Delta q_{A}}{C_{0}}=g_{L}V_{ds}\times \frac{\Delta q_{A}\left[AB\right]}{C_{0}}=g_{L}V_{ds}\times \frac{\Delta q_{A}}{C_{0}}Q_{max}\times \frac{\left[A\right]}{\left[A\right]+1/K}$$

where *g_L_* is the liquid gate transconductance, specifically, $g_{L}=g_{m}/\;V_{ds}$. [*A*] and [*AB*] represent the concentration of the analyte in the bulk solution and the surface density of the adsorbed analyte molecules, respectively. *q_A_* represents the charge of the analyte molecule adsorbed per unit of surface density on the carbon nanotubes. *C*_0_ represents the coupling constant between the analyte molecule and the surface of the carbon nanotubes. Details of the derivative process can be found in [92].

As demonstrated, the absolute current change is correlated with the voltage bias condition and the electrical characteristics of the CNT device.

3. Relative Response ($\frac{\Delta I}{I_{0}}$)

The response can be expressed as:

(6) $$Response=\frac{\Delta I}{I_{0}}=\frac{g_{m}\Delta V_{th}}{I_{ds0}}=\frac{g_{m}\Delta V_{th}}{g_{m}\left(V_{gs0}-V_{th0}\right)}=\frac{\Delta V_{th}}{\left(V_{gs0}-V_{th0}\right)}\;$$

Under a given operating condition, the gate voltage is constant, and the response uniformity in the linear region depends only on the uniformity of the device’s original *V_th_* distribution.

Considering Equations (2), (4), and (6), the response in the liner region is as follows:

(7) $$Response=\frac{Q_{max}}{4{log}_{10}e}\left[{log}_{10}c+{log}_{10}\left(e^{2}K\right)\right]\frac{1}{C_{ox}\left(V_{gs0}-V_{th0}\right)}\;$$

From the above equation, we can see that, in the linear region, the response of the sensor is linearly proportional to the logarithm (concentration).

In the subthreshold voltage regime, where *V_gs_* < *V_th_*, the subthreshold swing (SS) occurs: $\mathrm{SS}=\frac{d\left(V_{gs}\right)}{d\log_{10}(I_{ds})}=\frac{I_{ds}d\left(V_{gs}\right)}{d(I_{ds)}}\ln10\approx \frac{I_{ds}\Delta \left(V_{gs}\right)}{\Delta \left(I_{ds}\right)}\ln10$. The relative response is derived as $\frac{\Delta I_{ds}}{I_{ds0}}=\frac{\Delta \left(V_{th}\right)}{SS}\ln10$. Thus, the uniformity of the sensor response mainly depends on the SS. In addition, with a smaller SS, a larger response will be generated.

Above all, the use of a relative response can render the sensing performance less dependent on the transistor’s parameters and simplify the data acquisition system. Therefore, we believe that the definition of a relative response should be used more frequently in future practical applications. However, the development of a method with which to further improve the uniformity of the relative response is one of the future research directions for device performance optimization and sensor data processing.

## 5. Surface Functionalization for Biosensing

The detection of various biomolecules using CNTFETs is achieved by functionalizing the FET devices’ surface with specific biological receptors through non-covalent and covalent conjugation methods [95,96]. The functionalization process on CNT FETs using different biochemical molecules and chemical treatments is not only essential for constructing reliable biosensors, but it also plays a vital role in surface passivation so as to avoid unwanted, nonspecific binding to achieve high sensitivity and selectivity in high ionic and complex biofluid backgrounds, which will be discussed in the following sections [97].

**Figure 7:**
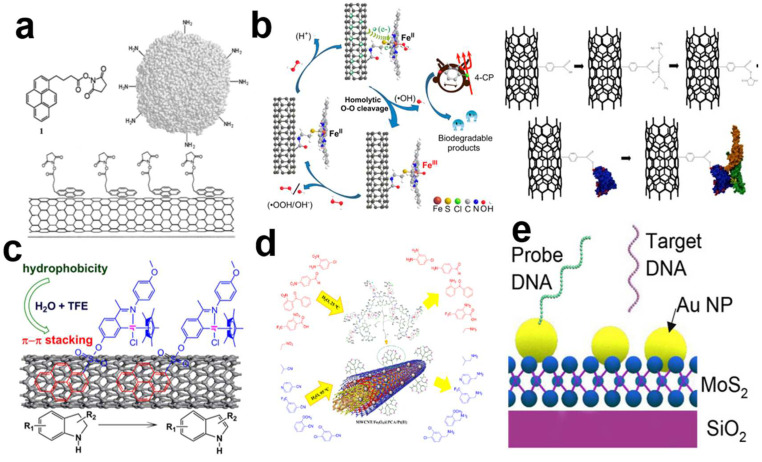
Functionalization of carbon nanotubes (CNTs). (**a**) Noncovalent modifications can be used to anchor molecules around nanotubes [102]. (Copyright © 2001, Journal of the American Chemical Society.) (**b**) The introduction of the -COOH group and –COOH to the surface of the CNT by the oxidation reaction [104,105]. The use of diazonium salt chemically functionalized MWCNTs to connect genetically engineered single-chain variable fragment (scFv) proteins with high OPN binding affinity to carbon nanotube field effect transistors [76]. (Copyright © 2017, Industrial & Engineering Chemistry Research; copyright © 2012, Journal of the American Chemical Society.) (**c**) A metal iridium complex catalyst was coated on the surface of CNTs through non-covalent bond accumulation [106]. (Copyright © 2017, Industrial & Engineering Chemistry Research.) (**d**) Functionalization process of CNTs for selective detection [105]. (Copyright © 2017, Industrial & Engineering Chemistry Research.) (**e**) MoS_2_ was functionalized with gold nanoparticles (Au NPs) of an optimized size and at an ideal density [103]. (Copyright © 2019, Nano Letters.).

Non-covalent functionalization is usually used in the direct modification of bare carbon nanotubes by intermolecular interactions, including hydrogen bonds and 𝜋–𝜋 bond interactions (using aromatic compounds or polymers), as shown in Figure 7a,c,d. The aromatic molecules, for instance, DNA or the 1-pyrenebutanoic acid succinimidyl ester, can interact with the CNT lattice through 𝜋–𝜋 stacking interactions, which are much stronger than van der Waals forces [12,90,98,99,100,101]. The main advantage of non-covalent functionalization is that it can preserve the electrical performance of CNTs to the greatest extent without damaging the CNT lattice [12,102]. However, the non-covalently functionalized receptor is weakly anchored to the surface, making it unsuitable for long-term and stable applications in harsh physiological environments and resulting in non-uniform sensor responses [103]. Furthermore, the modification process of non-covalent functionalization is random, leading to poor modification uniformity and the poor reproducibility of sensor performance. In addition, this modification process is not valid for functionalization on polymer-sorted semiconducting CNTs, which are the most mature CNT materials for bio-electronics, as discussed previously.

For comparison, the covalent modification of CNTs through certain defect sites or by dangling bonds on the surfaces or ends of tubes is more stable and reliable [100,107,108,109,110,111,112]. As shown in Figure 7b, Ni [104] and Rezaie [105] et al. introduced -COOH or the -COOH group to the surface of CNTs through oxidation reactions and then linked different ligands to achieve CNT biofunctionalization. However, the direct covalent linkage to the CNTs’ surfaces may damage their intrinsic electrical properties and result in inferior sensor performance [76,113,114].

In order to achieve maximum sensitivity and retain the inherent advantages of CNTs, a high κ dielectric connection layer can be introduced on the CNT surface to improve coupling with the receptor (Figure 7e) [115,116]. Au nanoparticles are always used as an extra linkage layer due to the abundance of –HS in biomolecules, and the reaction between Au with –HS is efficient and stable, thus providing a reliable, uniform, and universal functionalization surface. However, the gold nanoparticles will also introduce a shielding effect on the gate’s electric field. The abundance of active sites and the well-developed surface chemistry of the oxide dielectric layer in the FG structure provide a reliable and controllable surface for the covalent modification process. Highly sensitive and selective sensors mainly require that the biometric elements are fixed on solid surfaces with high density and a controlled orientation while maintaining the integrity and activity of the biomolecules in order to provide good spatial accessibility to the active binding sites of affinity ligands, while limiting non-specific adsorption and increasing stability [117,118]. In addition, the execution of a modification process to block the surface is also crucial for the inhibition of non-specific adsorption [119,120,121,122].

## 6. Performance Index Requirements for the Commercialization of Biosensors

When evaluating biosensors, factors such as selectivity, calibration range, linearity, precision, accuracy, and detection limits should be considered. Table 2 summarizes the figure of metrics most relevant to the biosensor. [123,124,125,126,127] The lower the limit of detection (LOD) and the more sensitive the detection of specific biomarkers, the earlier the disease can be detected by biosensors, while sensitivity and stability must be given proper weights. Advances in system integration and electrical interfaces have reduced the stringent requirements for stability. For example, electronic signals can be isolated and amplified, and trace analytes can be captured and released using pre-concentrators. Selectivity is the basis for the practical application of biosensors, and specificity is the limit of selectivity. Since the transfer properties of Bio–CNTFETs are very sensitive to their environment, which also means that they may respond to any type of analyte in the environment, the selectivity of the sensor needs to be considered. The combination of several highly selective and cross-selective sensing channels may lead to the optimal performance of a sensor array.

For field applications, especially with respect to disposable sensors, Bio–CNTFETs must be calibrated, and each sensor must provide a clearly defined response. The calibration curve is a plot of how the instrument response (the so-called analytical signal) changes as a function of the concentration. A plot of the instrument response versus concentration obtained by analyzing a series of standard solutions close to the expected analyte concentration in an unknown sample will show a linear relationship. Then, we obtain the concentration of the unknown sample.

In clinical practice, multiple biomarkers are often quantified from the same biological sample in order to gather sufficient information to make a diagnosis. Multiplexing requires a technology that can be developed to aggregate multiple sensing arrays so that standard solutions, negative controls and samples, and all the replicates of each biomarker can be analyzed. A Bio–CNTFET provides real-time and sensitive label-free bioelectronic detection and substantial redundancy in nanosensor arrays.

## 7. Biosensor Packaging

Packging is crucial to the successful application of biosensor chips in practical tests. Liquid solutions such as water, ion buffer, blood, urine, saliva, or tears are usually incorporated as the background medium in biosensor detection [128]. These aqueous solutions are not allowed to touch the metal interconnects as they will cause an electric short. Biosensor packaging is designed to introduce liquid analytes into the sensing interface through a miniature open chamber while maintaining the electrical integrity of the sensor and providing an interface to the user. The most important feat that a FET biosensor packaging must achieve is adequate electrical isolation between the liquid and metal interconnects in an extremely small area. Wire bonding and damming with epoxy are always used. To maintain their performance, Bio–CNTFETs must be packaged at low temperatures during the wire-bonding and glue-sealing processes, as high temperatures will denature the bio-affinity layer on the biosensor chip. Non-biocompatible materials may interfere with biological molecules; therefore, materials in close contact with biological materials must be biocompatible. To achieve high sensitivity and low interference, the system packaging and transmission lines must be optimized to minimize stray capacitance and inductance. With the continuous development and maturity of Bio–CNTFETs, researchers gradually tend to combine them with silicon-based electronic devices to standardize, improve, and strengthen their sensing control and performance, and to develop low-cost, low-noise, portable electronic biosensors [129,130].

**Figure 8:**
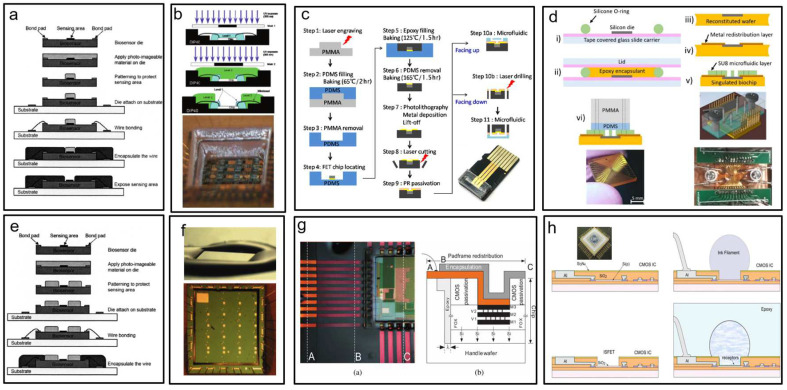
Current, more feasible biosensor packaging technology. (**a**) Photolithography defines the package protection layer and leaves contact holes for the sensing area on the packaged biosensor module [131]. (Copyright © 2005, In Proceedings of the 2005 7th Electronic Packaging Technology Conference.) (**b**) The high-aspect-ratio patterning of the photocurable material; the bonding wire is encapsulated with two layers of Loctite [132]. (Copyright © 2009, In Proceedings of the SENSORS, 2009 IEEE.) (**c**) The process steps of FET-based biosensor packaging, including microfluidic channels [133]. (Copyright © 2017, ECS Journal of Solid-State Science and Technology.) (**d**) Schematic diagram of laboratory-scale fan-out wafer-level packaging process [134]. (Copyright © 2018, Sensors and Actuators.) (**e**) Photolithography defines the SU-8 dam as a barrier [131]. (Copyright © 2009, In Proceedings of the SENSORS, 2009 IEEE.) (**f**) Use of SU-8 dam and halting at the dam by the surface tension of epoxy resin [132]. (Copyright © 2009, In Proceedings of the SENSORS, 2009 IEEE.) (**g**) Use of micromechanical redistribution pad frame (MRP) to package the chip, place the chip in the cavity, and rewire the pad frame, so as to realize the integration of microfluidics [132]. (Copyright © 2009, In Proceedings of the SENSORS, 2009 IEEE.) (**h**) Direct ink writing (DIW) used to form a microfluidic system (MFS) can quickly package and produce integrated circuits with sensors [135]. (Copyright © 2017, Materials Science in Semiconductor Processing.)

Fan et al. [131] coated a wafer with a photo-imageable material and used lithography to create a protective layer on the sensor’s sensing area. The protective layer was removed after encapsulation, leaving contact holes on the biosensor module in the sensing area (Figure 8a). Dandin et al. [132] created a high-aspect-ratio structural opening on a sensor chip via lithography (Figure 8b). However, the encapsulated resin was partially dissolved in ethanol and swelled in an ionic solution, resulting in shedding and more residue. As shown in Figure 8c, Hsu et al. [133] used coplanar technology to fabricate an FET biosensor embedded in an FET chip with the same surface plane. Electrical interconnection was achieved by a lift-off process across the boundary between the chip and the plastic substrate. It was then passivated with a photoresist to open the source-drain channel and gate electrode, which were then combined with a microfluidic channel made of PMMA. The technology has the advantages of low cost, a simple manufacturing process, and high flexibility with respect to chip positioning. As shown in Figure 6d, Laplatine et al. [134] used fancy-out wafer level packaging (FOWLP) technology to package a single chip into an epoxy resin substrate. The chip and the bonding pad were interconnected in a fan-out fashion by a stripping technique to create a metal redistribution layer (RDL). The SU-8 dam was designed using lithography, and the surface tension of the epoxy resin was used to stop and cure the covering wire at the dam (Figure 8e,f) [132]. In addition to correct flow characteristics and surface tension, the requirements for epoxy resins include biocompatibility, a sufficiently fast curing time, and good adhesion to the chip surface. Dandin et al. [132] fixed the chip into a silicon cavity using epoxy resin and coated SU-8 to fill the void (Figure 8g). The planar metal leads were then designed by lithography and passivated using SU-8. The micromechanical redistributed pad frame (MRP) method requires direct contact with the chip’s surface, with the aim of allowing late microfluidic integration. Gubanova et al. [135] used the sacrificial layer technique to form microfluidic systems (MFS) on the chip’s surface by direct ink writing (DIW) (Figure 8h). The DIW used to form MFS enables rapid transition to large-scale production, thereby enabling a rapid, automated, and autonomous process.

With the gradual expansion of biosensors’ functions from routine biochemical analysis to in vivo assays, multi-index assays, and online assays, the corresponding application fields are rapidly expanding. These issues should be investigated and addressed in parallel with the expansion of the development of new nanomaterials for biosensors. Once these engineering aspects are systematically addressed, we anticipate that ongoing academic research will be implemented in commercially viable prototypes by the industry in the near future.

## 8. Bio–CNTFET Applications

Carbon nanotube FET transducers detect biologically significant molecules such as proteins, enzymes, antibodies, viruses, and DNA by modifying their surfaces with specific molecules (recognition elements) [31,136,137,138,139,140]. The recognition elements can bind specifically to the target, causing physical and/or chemical changes that alter the electronic signal of the CNTFET. CNTFET biosensors have various applications, depending on the analyte, including metal ions [115,128], hormones [141], protein sensors, DNA sensors, bacterial sensors, and cell sensors. Bio–CNTFETs have been proven to be ultra-sensitive biosensors, with applications in aptamer sensors [115,128,142] and label-free protein sensors [143,144,145]. In this section, we will review recent developments in Bio–CNTFET Technology.

**Figure 9:**
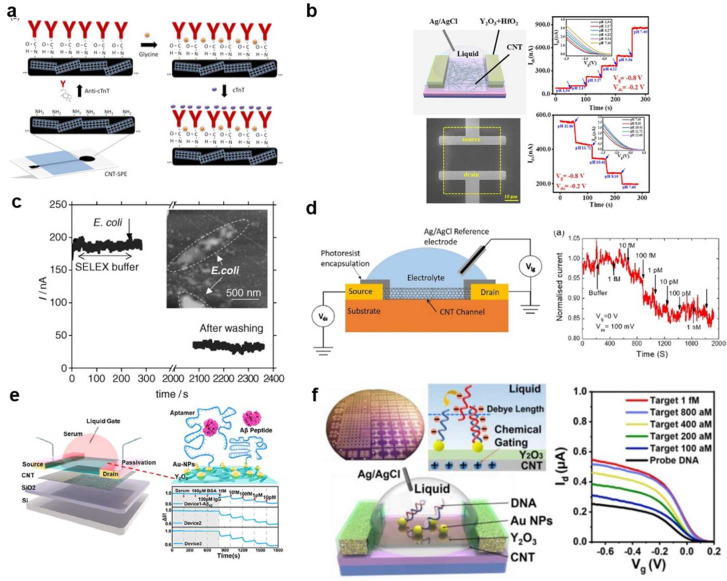
Bio–CNTFETs have been used in the diagnosis of viruses, ions, proteins, antibodies, and cells. (**a**) Detection of protein [146]. (Copyright © 2013, Talanta.) (**b**) Monitor the PH of the solution [148]. (Copyright © 2023, Carbon.) (**c**) The detection of biological cells [147]. (Copyright © 2008, Small 2008.) (**d**) The selective detection of SARS-CoV-2 [149]. (Copyright © 2021, A proof-of-concept study. Materials Today: Proceedings.) (**e**) Early diagnosis and large-scale screening of AD [56]. (Copyright © 2022, ACS sensors.) (**f**) The detection of specific DNA sequences and MVs [90]. (Copyright © 2020, ACS nano.)

Silva et al. reported [146] that CNTs can be functionalized with specific antibodies to detect different proteins, as shown in Figure 9a. The binding of proteins to CNTs through receptors on the surface leads to changes in the source leakage current and voltage. As demonstrated by So et al. [147], CNTs can also be functionalized with specific antibodies to detect different cells, such as bacteria, pathogenic yeast, or mammalian cells (Figure 9c). Wang et al. [148] reported that CNTFET-based biosensors can be used to monitor the PH of a solution (Figure 9b). Virus detection typically involves either detecting the DNA of the virus through immobilizing DNA or RNA, or directly detecting the virus by immobilizing antibodies or peptides.

The COVID-19 pandemic has highlighted the importance of sensitive, specific, and rapid diagnostic tests. Quick detection not only enables effective treatment but also helps prevent the spread of infectious diseases [19,150]. Although the use of a polymerase chain reaction (qPCR) is a common detection method, CNTFET electronic biosensors provide a faster alternative by eliminating the time-consuming amplification steps of qPCR. As reported by Thanihaichelvan et al. [149], a reverse sequence of the RNA–dependent RNA polymerase gene (RdRP) of SARS-CoV-2 was used as a probe in a liquid-gated CNT network FET biosensor, resulting in the selective detection of SARS-CoV-2, as shown in Figure 9d. Chen et al. [56] (Figure 9e) reported that the large-scale production of a semiconductor CNTFET biosensor achieved high sensitivity and selectivity for detecting the AD core blood biomarker of β-amyloid (Aβ), offering a low-cost and rapid method for the early diagnosis and large–scale screening of AD. In [90], Liang et al. reported (Figure 9f) a floating gate type CNTFET biosensor for the selective and quantitative detection of specific DNA sequences and MVs, and this biosensor has the potential to become a general biosensing platform. By changing different bio-probe molecule, selective detection of different disease markers can be achieved.

Bio–CNTFETs are recognized as one of the most effective platforms for electronic biosensors [151,152]. They have the ability to selectively detect metal ions [115] and various biomolecules such as hormones [141], viruses, and whole cells. The ability of Bio–CNTFETs to detect low-concentration biomarkers without the need for labeling is expected to provide new insights into the roles of various biomarkers in the etiology of specific diseases and create new opportunities for medical diagnosis. The market availability of CNTFET biosensors is increasing steadily. Due to their good sensitivity, low cost, and low power consumption, CNTFET biosensors have been widely used in the medicine, environmental monitoring, and security. In addition, the programmability of CNTFET biosensors has also enabled them to be widely used in the intelligent home, intelligent transportation, and intelligent manufacturing fields.

## 9. Bio–CNTFET Problem

**Figure 10:**
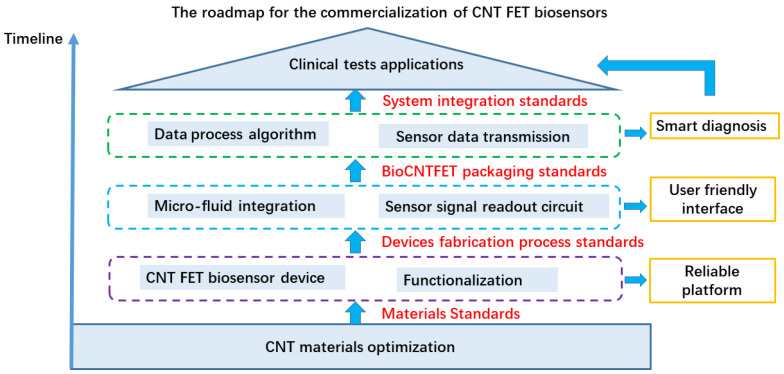
Schematic representation of the carbon nanotube-based biosensor field effect transistor industrialization process.

The high sensitivity of Bio–CNTFETs makes them a promising platform for electronic biosensors. However, their wide application is limited by the accuracy and reliability of their sensing results. In order for biosensors to be valuable in both research and commercial settings, they must be able to identify target molecules, have suitable biometric components, and have the potential to be more widely used compared to current detection technologies (Figure 10). The following engineering issues need to be addressed in order to commercialize Bio–CNTFETs as biosensors:

1. The optimization of the device structure to reduce fluctuation and improve its signal-to-noise ratio (SNR), stability (caused by baseline drift in complex storage environment), and sustainability.
2. The development of a controlled surface Bio–functionalization process for multiple-target detection. At present, the use of silicon nanowires for detection has proven reliable, and there are very few available examples based on CNTs. Multiplex detection is particularly attractive in medical diagnostic scenarios, where more than one analyte can be used as a biomarker for a single disease state. For example, nanomaterials should be integrated with micro biochips (labs on a chip) for sample processing and analysis for multiple clinical diagnosis.
3. The improvement of the reproducibility and affordability of large-scale manufacturing.
4. The development of reliable, low-cost packing technology.
5. The addressal of the challenge of sensor calibration for various applications.
6. The simplification of user interventions.
7. The establishment of standard performance indicators of product quality.
8. The performance of extensive clinical testing to prove the reliability and safety of the product.
9. The addressal of the lifetime and baseline drift of the devices in complex storage environments, as well as the challenge of detecting multiple biomarkers for a single disease.

Among the many challenges, the consistency and reproducibility of CNT FET biosensor devices’ performance is one of the most important challenges, as this plays a decisive role in the reliability and accuracy of the results. Therefore, every process—including materials synthesis, device fabrication, and biomodification—needs to be handled carefully (Figure 10). Another challenge is system integration (Figure 10), as current measurements have mainly been performed in ideal conditions such as pure buffer solutions, whereas real physiological samples are much more complex and can introduce interference and contamination effects.

In addition, the development of parallel and integrated biosensor arrays with specific functions through spatially resolved surface functionalization for multiplexing is still a challenge. However, Bio–CNTFETs offer several significant advantages over traditional methods such as PCR, mass spectrometry, and enzyme-linked immunosorbent assays (ELISAs), including simplicity, low cost, portability, ultra-high sensitivity, excellent selectivity, and label-free, real-time electrical detection. As these technical issues are addressed, it is expected that academic research will translate into commercially viable prototypes in the near future.

The future of CNTFET biosensors is promising, with opportunities for the development of multi-targets and multi-functional arrays and the integration of microfluidic and CNT signal process circuits for self-adaptive disease diagnosis. The low-temperature fabrication process of CNTs enables low-effort 3D integration and the high-throughput processing of sensing data [153]. These smart microsystems have the potential to revolutionize the way we diagnose and manage diseases.

## 10. Conclusions

In conclusion, Bio–CNTFETs have the potential to revolutionize the field of biosensing with their high detection speed, sensitivity, and selectivity, allowing for single-molecule-level biological detection. The development of Bio–CNTFET technology is the focus of this article, and the main challenges that need to be overcome for its widespread deployment are outlined. The most crucial factor for the large-scale deployment of Bio–CNTFETs is the reproducibility and stability of the sensor response. With continued efforts towards optimizing CNT materials, device configurations, fabrication processes, functionalization, and packaging, along with the continuous advancement of clinical testing, it is believed that Bio–CNTFET technology will soon be adopted by the industry. The future of Bio–CNTFETs is promising, as the application market matters, with opportunities to develop multi-target and multifunctional arrays and integrate microfluidic and CNT signal-processing circuits for adaptive disease diagnosis.

## Figures and Tables

**Table 1:** Bio–CNTFET-based detection of various biomarkers.

Type of CNT	Bioreceptors	Target Biomolecules	LOD	Sensitivity	Refs.
single carbon nanotubes	streptavidin ligand	biotin	-	-	[48]
	an enzyme activity sensor	Glucose oxidase	0.1 mM	-	[50]
aligned CNT sensor array	t-tau p-tau_181_	AD biomarkers	(Aβ_42_) 2.13 fM, (Aβ_40_) 2.20 fM, (t-tau) 2.45 fM, (p-tau_181_) 2.72 fM	-	[51]
	IgE aptamer	human immunoglobulin E (IgE)	16 nM	52 nM	[52]
	DNA T	DNA probe	2 pM	-	[53]
CNT network film	DNA-probe	miRNA	0.87 aM	-	[54]
	DNA-probe	DNA	1 pM	-	[55]
	β-amyloid	Aβ aptamer	(Aβ_42_) 42 aM(Aβ_40_) 55 aM	1 fM	[56]

**Table 2:** Figures of merit for performance assessment of biosensors.

Figure of Merit	Definition
Sensitivity	Sensitivity is a critical parameter in characterizing the static behavior of a sensor. It is typically defined as the ratio of the output change to the input stimulus that caused it. This measure reflects the ratio of the signal change to the noise that the sensor can discern. In addition, sensitivity also indicates the sensor’s capability to detect the lowest concentration of target biomolecules while still being able to distinguish noise. Sensitivity is the slope of the calibration or dose curve used in analysis.
Selectivity	The selectivity of a biosensor refers to its ability to detect the target analyte despite the presence of similar analytes and contaminants. Selectivity can be quantified using the ratio of a biosensor’s response to the target analyte to its response to a similar analyte at a given concentration. Biosensors with high selectivity have low cross-reactivity with other molecules, enabling precise and dependable detection of the target analyte.
Specificity	This is the ultimate limit of selectivity and applies only to a method/sensor that is capable of exclusively detecting the analyte, without suffering from any interference (100% selectivity).
Limit of detection (LOD)	Detection limit is used to evaluate the lowest concentration of the analyte in a biological sample at which a specific biomarker can be detected, but with no guarantee of precision. It can be calculated by considering a signal y_LOD_ = y_c_ + k × S_c_, where yc is the mean signal for control experiment, S_c_ is its standard deviation, and k is a numerical factor referring to the chosen confidence level (generally, 3).
Limit of quantification (LOQ)	The lowest concentration of an analyte that can be accurately quantified or measured with a certain degree of precision and accuracy by an analytical method or instrument.
Dynamic range	The "concentration range" refers to the range of analyte concentrations that cause a change in the output signal.
Repeatability	"The degree of scatter" is a measure of the variability or dispersion of data obtained from multiple measurements of a specific parameter under the same set of operating conditions. It is an important consideration in evaluating the precision and reproducibility of experimental results.
Reproducibility	"The degree of scatter" refers to the variability or dispersion of data obtained from multiple measurements of a specific parameter under different operating conditions. This is an important factor to consider when evaluating the precision and reproducibility of experimental results, as it provides insight into the level of measurement error and the potential impact of environmental or technical factors on the data.
Linearity	Refers to the degree to which the actual relationship curve between sensor output and input deviates from the fitted straight line.
Uniformity	This means that the initial performance of different sensors is highly similar, and the difference between devices is very small.

## Data Availability

Data Sharing not applicable.
